# Bibliographical Notices

**Published:** 1838-07-04

**Authors:** 


### BIBLIOGRAPHICAL NOTICES.

Nature and Treatment of Diseases of the Ear. By Dr. William Kramer. *Second Edition of the Author's Treatise on Chronic Deafness, much improved and enlarged. Translated from the German with the latest improvements of the author since the last London* [?] *edition*, by James Risdon Bennett, M. D., Member of the Royal College of Physicians of London, &c. &c. &c. Philadelphia: Thomas, Cowperthwait & Co. 1838. 8vo. pp. 250.

(Continued from page 209.)

Affections of the cavity of the tympanum and the Eustachian tube, Dr. Kramer classes under "*Diseases of the middle Ear*," and as those distinctly recognised are inflammation of the Eustachian tube, and of the cavity of the tympanum with that of the subjacent cellular membrane, these are alone treated of.

Before entering into their consideration, our author devotes a chapter to a highly important but neglected part of his subject—*catheterism* of the Eustachian tube; and, as with the exception of Deleau, no aurist who has yet written appears to possess any practical acquaintance with it, we shall present our author's views with some copiousness.

The inferior angle of the opening of the tube lies a little deeper than the inferior nasal meatus, looking obliquely downwards and backwards. The catheter should consequently be introduced into the *inferior* nasal meatus, and pushed carefully but rapidly forwards over the bottom of the fossa into the top of the pharynx, when the beak of the instrument is to be depressed, and at the same time rotated a quarter of an inch upon its axis, by which it is pushed into the mouth of the canal. We shall not here describe the apparatus for retaining the catheter in the tube during its use, which is a modification of Itard's. The catheter employed by Dr. K. is of silver, inflexible, six inches in length, and the caliber varying in size, from that of a crow-quill to that of a goose-quill. The extremity is well rounded and curved "to the distance of five lines from the further extremity, exactly at an angle of 144°." (p. 160.) The caliber is the same throughout the tube, whose proximal end terminates in a funnel-shaped dilatation, six inches long, to admit the pipe of the syringe. A ring is attached to this portion, on the same level with the beak, by means of which, the situation of the latter, when out of sight, may be known. The catheter is graduated to inches.

By this contrivance, Wathen, Douglas, Saissy, Itard, and others, recommended and practised the injection of tepid water into the cavity of the tympanum, as a diagnostic as well as therapeutic means. To Deleau is commonly awarded the credit of being the first to perceive the inconveniences of the aqueous injections, and to substitute air for water. Clelland, an English army surgeon, more than a century since, in a paper published in the Philosophical Transactions, appears to have recommended the same procedure, although no evidence is offered of his having actually practised it. Speaking of catheterism of the Eustachian tube, of the syringe he says—"or they will admit to blow into the Eustachian tube, and so force the air into the barrel of the ear, and dilate the tube sufficiently for the discharge of the excrementitious matter that may be lodged there." (*Phil. Trans.*) Deleau kept the construction of his apparatus a secret, but Dr. Kramer describes and figures an air press, which he pronounces to prove equal to every exigency. Auscultation, through the agency of the air-douche, is employed as a valuable aid in aural diagnosis. The operator placing his own ear close to that of the patient, which is the subject of examination, opens the cock of the machine, and listens to the sound elicited by its entrance into the cavity of the tympanum.

"When the first shock of this powerful stream is over, or if it has not been so powerful, there is heard from the continued stream of air rushing into the ear, a blowing and rustling, which appear to issue from the external meatus, and fill the whole ear of the patient. All deviations from this noise (the peculiarity of which can be rendered clear and comprehensible only by repeated observation) are morbid, and afford very certain conclusions as to the particular diseased changes in the organic and functional condition of the ear." (p. 188.)

When the tube is impervious to air, Dr. K. employs catgut bougies to remove the obstruction.

*Inflammation of the mucous membrane of the middle ear with accumulation of mucus*, is produced by cold, and is analogous to the catarrhal affections of the adjacent tissues. The prognosis is favourable, even when the affection has been neglected, and has degenerated into the chronic state. General remedies are demanded when any constitutional disorder exists. The only certain curative procedure is to overcome the mucous accumulation of the tube, by acting through the pharyngeal opening, and for this purpose Dr. K. uses the air-douche. He condemns unequivocally all attempts to remove the obstruction, by injections through perforations of the mastoid process, or membrana tympani.

*Inflammation of the Eustachian tube with stricture*, as well as that with *obliteration of the canal*, Dr. Kramer regards as hopeless, nor does he advise perforation of the membrana tympani in such cases, from the mucous membrane of the tympanal cavity being also generally involved.

*Inflammation of the cellular tissue and periosteum in the cavity of the tympanum*, (*true internal inflammation of the ear*,) complicated as it is usually with cerebral disease, is a highly dangerous affection. Two forms are distinguished, the acute and chronic. In the cerebral disorder the ear is apt generally to be overlooked, particularly in young children. Whenever we have pain in the head occurring, associated with that of the ear, and a sensitive condition of the meatus, we should be induced to examine that organ carefully. The prognosis is, of course, by no means flattering, and the most energetic treatment is requisite to afford any chance of a successful issue.

Concerning the diseases of the internal ear, the most fanciful and speculative hypotheses have been indulged in. That organic disease of the labyrinth does occur cannot be doubted; but from our imperfect means of investigation it can only be guessed at. The only undoubted affection of the internal ear, according to our author, is functional derangement of the special nerve of the auditory apparatus—*nervous deafness.* The absence of any appreciable organic lesion in the ear, after complete local investigation by the means already mentioned, together with the objective symptoms, will enable us to decide with tolerable certainty on its existence.

"Debility of the auditory nerve appears under two forms, differing essentially from each other. 1. Attended by augmented irritability—erethismus. 2. With diminished irritability—torpor. Tinnitus forms the essential point of difference between the two. It belongs, without exception, to the erethitic form, whilst to the torpid form, it is altogether foreign." (p. 211.)

With the exception just stated, the symptoms are identical in the two forms of nervous deafness. The disease very generally commences insidiously, and some time elapses before the patient is aware of suffering any diminution of acuteness in his hearing. The plan of treatment proposed by Dr. Kramer, and which, in a measure, he is entitled to the credit of introducing to the profession, is a signal step in aural therapeutics, if subsequent experience in other hands, confirms the favourable results announced by him. Itard was the first to recommend the introduction of etherous vapor into the middle ear through the Eustachian tube. But from his imperfect diagnostic knowledge, (not being in the habit of investigating the Eustachian tube,) with consequent irresolution, he abandoned his method. Itard proposed to generate the vapour by dropping the acetous ether on an iron saucer made red hot. But a decomposition thus takes place, and an acrid irritating gas enters the ear, which, though beneficial in the torpid form of nervous deafness, is highly prejudicial in the erethitic variety.

By an apparatus invented by Dr. K., the ether is generated "at the ordinary temperature of the room." In the torpid form, where there is necessity for a more stimulating treatment, our author employs a modification of Itard's apparatus. Decided preference is to be given to the acetous ether. Ten cases are detailed, in all of which the patients experienced more or less unquestioned relief by perseverance in this plan of treatment, which should never be tried for a less period than three months.

The two concluding chapters are devoted to the consideration of "Ear Trumpets" and "Deaf Dumbness." After examining with some acuteness those instances reported of the restoration of deaf mutes to hearing, Dr. Kramer says:

"After this complete statement of all the accounts of deaf mutes that have been said to be cured, I may venture to declare, distinctly, that hitherto *no single deaf mute has been cured*—that is to say, *has been rendered capable of communicating, like a person who hears well, with his fellow-men, in an unrestrained manner, by means of hearing under all circumstances.*" (p. 250.)

In presenting to our readers this extended digest, we believe that we have rendered them an acceptable service. We have but little doubt that all of them will hasten to possess so truly valuable and essential a work. Dr. Kramer's descriptions of the various lesions of the ear are distinguished for clearness and fidelity. His experience with aural maladies has been very extensive, and his ability to treat of them is unquestioned. His reputation in Germany is high and well-earned. Dr. Bennett's translation is, as a whole, quite creditable.

---

*A Treatise on the Functional and Organic Diseases of the Uterus. From the French of* F. Duparque, Docteur en Medicine de Paris, &c. &c. *Translated, with notes*, by Joseph Warrington, M. D. of Philadelphia. Philadelphia: Desilver, Thomas & Co. 1837. 8vo. pp. 455.

The pathology and treatment of uterine diseases are still involved in much obscurity, nor do they obtain the attention which their importance demands. Within the last few years, indeed, our acquaintance with this class of affections has been somewhat extended; and for this, we are chiefly indebted to the French; especially Desormeaux, Dugés, Lisfranc, Mesdames Boivin and Lachapelle, &c. Yet the innumerable and distressing lesions of this vital organ of the female economy, are very imperfectly understood, and very indifferently managed. To our limited pathological knowledge may, we think, justly be attributed the repeated failure of our curative efforts. In our treatment of the disorders of the womb, a crude empiricism appears to have usurped the place of rational therapeutics, and promises, unless promptly met, and energetically attacked, to maintain, for some time, at least, its unhallowed position, to the opprobium of our art.

The introduction and application of the speculum in the investigation of the diseases of the womb, we look upon as one of the happiest events in modern medicine, promising, as it does, to elucidate a highly intricate subject, increase our information, and ameliorate suffering. The necessity of direct exploration, by means of the touch, has long been universally conceded; and that by the eye, is now acknowledged, by every enlightened physician, as still more momentous. The volume, form, and consistence of the organ may, to a certain extent, be appreciated by tact; the intrinsic alterations of colour and texture, can alone be properly revealed by ocular examination. By this means we can detect the earliest departure from the natural condition, and thus are enabled to arrest the disease in the initial stage, and curtail sensibly the amount of misery.

No culpable fastidiousness of the practitioner, or false delicacy of the patient, should be allowed to interfere with the establishment of liberal and rational principles in this department of medicine, or with any attempt to rescue it from the reproachful abasement in which we now find it. So soon as the organic lesions of the uterus become the subject of actual perception and appreciation, a new impulse will be imparted to their study, and the same untiring zeal and commendable industry will characterize the observers in this, as well as the other branches of medical science; they will receive the minute attention and exact discrimination they demand, and precision in diagnosis, and certainty in therapeutics, will be the happy and inevitable result. We are solemnly convinced, ourselves, of the immense and peculiar practical advantages of the speculum uteri, and for which no other known method can be substituted. We urge, then, confidently and earnestly upon the consideration of the profession the propriety of giving their serious attention to this means of exploration, now too generally neglected, or had recourse to as a last resort. In doing so, we are abundantly aware of the opposition we shall encounter, and the strong prejudices we have to combat. No one can be more solicitous than ourselves to preserve, in all its purity, that respect to female delicacy which should invariably distinguish our professional intercourse with the sex; but we candidly confess, that we cannot understand, much less appreciate, either the justness or wisdom of that spirit which, from mistaken refinement, would sacrifice life, or permit it to linger in misery. Every thing in such cases, we feel assured, depends upon the physician; and that if by propriety of conduct he merits confidence, he will readily obtain it, and thus dissipate irrational prejudice.

We would not limit the employment of the speculum to the more formidable diseases of the uterus, where it can do but little more than assist our diagnosis, and confirm the futility of all curative aid. It is in the more common affections of the womb, in general so obscure and perplexing, that we counsel its assistance. How frequently does a simple mucous vaginal discharge baffle, year after year, the best directed resources of our art, becoming the pest of female existence—converting the life of the matron into a state of constant loneliness and despondency, by wrecking all the hopes of maternity, and rendering that of the maiden one of solicitude and gloom.* This is no overcharged picture, but one of daily realization. To remove the disease, the cause must be recognised, and this can be accomplished only by the means just pointed out—actual examination of the organ in its diseased state.

The work whose title stands at the head of this article, has now been before the public for some time, and been received with general and deserved approbation. Our object in noticing it is not, then, for the sake of novelty, but from the conviction that its perusal will go far towards contributing to the dissemination of sound pathological and therapeutic views. Our thanks are due to Dr. Warrington, for placing it within reach of the American reader.

---

INSTITUTES OF SURGERY: *Arranged in the order of the Lectures delivered in the University of Edinburgh.* By CHARLES BELL, K.G.H.F.R.SS.L.&E., Professor of Surgery in the University of Edinburgh, &c. &c. &c. In two volumes. Vol. II. Edinburgh. MDCCCXXXVIII. 12mo. pp. 380.

THE unfavourable opinion we ventured to express, in a previous number,* of the Institutes of Surgery, on the appearance of the first volume, has been confirmed by a careful perusal of the second and concluding one, just issued. We then confessed that the source had led us to expect, naturally, we thought, a production which would, at least, sustain, if not increase the author's reputation; but had the anticipations indulged in, been of the most negative nature, they would have been lamentably disappointed.

In the present volume there is a repetition of all the blemishes which disfigured its predecessor, without the redeeming chapters. We have the same dogmatical tone; the same hasty, incorrect generalization; the same wholesale criticism on persons as well as things, settling in a word, and very often by the more summary process of a note of interrogation, the most intricate and dubious points of surgery. The same random and flippant tone, and offensive affectation of smartness, pervade the whole of these pages. Indeed, the reader of the Institutes should possess the most accommodating and easy disposition, the most absolute faith, and veneration for authority. He should never allow his curiosity to demand more knowledge than is compressed in the volume before him, or be unreasonable enough to ask the wherefore, or allow an impertinent doubt to intrude as to the truth of what is laid down. All honest spirit of inquiry must be repressed—the professional sanctity of the

* Leucorrhœa has long been considered one of the most common causes of barrenness in the female. The recent microscopal experiments of Donné, by which he found the animalculi of the semen masculinum, destroyed by certain mucous discharges of the uterus and vagina, would seem to throw some light upon the cause.

* Medical Examiner, No. V. p. 77.

author must not be invaded, or his infallibility questioned.

Our author never condescends to explain, but cuts the Gordian knot, by announcing the fact, supported by his own indisputable mandate. That in two volumes of nearly eight hundred pages, coming from one whose celebrity is undoubted and deserved, there are occasional remarks of value, it would be preposterous to deny; but these vastly resemble the migrations of certain ethereal spirits to this mundane sphere, and are deplorably infrequent. Indeed, Sir Charles Bell seems to be aware that particular paragraphs are endowed with superior merit, or else feared that amid the general effulgence of wisdom they would be slighted; for we have almost every page embellished with divers ☞, thus causing it marvellously to resemble the cross-roads of some recently discovered country. This we were at first sight disposed to regard as evidence of the capricious fancy of the typographical imp, but which further acquaintance and more mature deliberation convinced us to arise from the author's consideration for his reader's time, they serving as guides to the oases in this mighty maze of inanity and pretension. The unwary, who, from veneration or curiosity, have been trapped into a perusal of the work, will doubtless regret that Sir Charles' charity did not extend further, by stamping a large ☞ on the cover with *noli me tangere* legibly inscribed thereon.

We cannot conclude without reference to the chapter on syphilis, the style of which is a very fair sample of that of the whole work. The commencement is characteristic.

"Is there any experienced senior of the profession, who, having a son of eighteen or twenty, and that son having a chancre, that would treat him without mercury? No! there is not such an unnatural person." (p. 228.)

Rather let it be asked, who would? Indeed, the whole of Sir Charles Bell's views on the subject are of the most antiquated description, and pernicious tendency. He tells us that

"The primary ulcer (chancre) is the same to-day with that described by Mr. Hunter,—the same with that described three hundred years ago. (What is the conclusion?") p. 231.

This statement is one now universally conceded to be erroneous,—the appearance assumed by the sore varying with its location. A page or two subsequent contains the following paragraph, which sufficiently condemns the indiscriminate and sweeping practice just recommended.

"☞ See that you distinguish a tear of the root of the frenum; it very much resembles a chancre in an early stage. When the chancre is really here, it produces violent inflammation [?] Remember, too, that the glans and prepuce, are peculiarly exposed to excoriation and ulcer not venereal, and that if you give mercury for these, you enter a labyrinth." (p. 232.)

The justness of the above advice is indisputable; but our author does not deign to point out any means of diagnosticating the true from the spurious disease; nor are we aware of any that can be implicitly relied on. He repeats his caution at p. 239.

"Before you place your patient on a course of mercury, take especial care that the case is made out, and that there is no doubt of the nature of the disease."

We join cordially Sir Charles Bell in his condemnation of the application of caustic in the treatment of chancres, having been so often ourselves the witness of the irreparable injury it entails. We deem it particularly necessary at this time to repeat this caution, from the countenance the practice would seem to obtain from the respectable authority of Mr. Ricord, on the ground of a specific virus contaminating the system, a point by no means yet satisfactorily established.

